# Stable Isotope Metabolic Labeling with a Novel ^15^N-Enriched Bacteria Diet for Improved Proteomic Analyses of Mouse Models for Psychopathologies

**DOI:** 10.1371/journal.pone.0007821

**Published:** 2009-11-13

**Authors:** Elisabeth Frank, Melanie S. Kessler, Michaela D. Filiou, Yaoyang Zhang, Giuseppina Maccarrone, Stefan Reckow, Mirjam Bunck, Hermann Heumann, Christoph W. Turck, Rainer Landgraf, Boris Hambsch

**Affiliations:** 1 Max Planck Institute of Psychiatry, Munich, Germany; 2 Schizophrenia Research Institute, School of Health Sciences, University of Wollongong, Wollongong, Australia; 3 CNS Research, F. Hoffmann-La Roche Ltd., Basel, Switzerland; 4 Affectis Pharmaceuticals AG, Martinsried, Germany; 5 Max Planck Institute of Biochemistry, Martinsried, Germany; 6 Silantes GmbH, Munich, Germany; L'université Pierre et Marie Curie, France

## Abstract

The identification of differentially regulated proteins in animal models of psychiatric diseases is essential for a comprehensive analysis of associated psychopathological processes. Mass spectrometry is the most relevant method for analyzing differences in protein expression of tissue and body fluid proteomes. However, standardization of sample handling and sample-to-sample variability are problematic. Stable isotope metabolic labeling of a proteome represents the gold standard for quantitative mass spectrometry analysis. The simultaneous processing of a mixture of labeled and unlabeled samples allows a sensitive and accurate comparative analysis between the respective proteomes. Here, we describe a cost-effective feeding protocol based on a newly developed ^15^N bacteria diet based on *Ralstonia eutropha* protein, which was applied to a mouse model for trait anxiety. Tissue from ^15^N-labeled vs. ^14^N-unlabeled mice was examined by mass spectrometry and differences in the expression of glyoxalase-1 (GLO1) and histidine triad nucleotide binding protein 2 (Hint2) proteins were correlated with the animals' psychopathological behaviors for methodological validation and proof of concept, respectively. Additionally, phenotyping unraveled an antidepressant-like effect of the incorporation of the stable isotope ^15^N into the proteome of highly anxious mice. This novel phenomenon is of considerable relevance to the metabolic labeling method and could provide an opportunity for the discovery of candidate proteins involved in depression-like behavior. The newly developed ^15^N bacteria diet provides researchers a novel tool to discover disease-relevant protein expression differences in mouse models using quantitative mass spectrometry.

## Introduction

The identification of candidate biomarkers as novel diagnostic tools and drug targets for psychopathologies is of great importance in neuropsychiatric research. While genes fundamentally shape physiology and pathophysiology, proteins are the final executive force of all cellular processes that finally drive physiology and behavior in health and pathology. Still, whole proteome approaches are scarce due to the complexity of differential protein analysis. Mass spectrometry (MS) is currently the most comprehensive method to analyze differences in protein expression and formation of the proteome. However, standardization of sample handling is problematic and sample-to-sample variability is difficult to control. For an accurate and sensitive comparative proteome analysis metabolic labeling of one sample with a stable isotope is the preferred approach. This method results in an enrichment of the stable isotope in every protein *in vivo*, which can be compared with an unlabeled proteome by combining the two samples prior to MS analysis.

Whereas the method of metabolic labeling with ^15^N has already been used decades ago [1], its application in brain research is just about to be realized by combining *in vivo* metabolic labeling of mammals with MS.

Metabolic labeling is well established for cultured cells (stable isotope labeling by amino acids in cell culture, SILAC, [2], [3]), plants (SILIP; hydroponic isotope labeling of entire plants, HILEP, [4]–[6]) and non-mammals including *D. melanogaster* and *C. elegans* [7]. Recently, the first successful labeling of rodents (Stable Isotope Labeling in Mammals, SILAM) and protein expression measurements were reported [7]–[10].

Wu et al. [8] showed for the first time that rats, when fed a ^15^N blue-green algae diet from weaning onwards, sufficiently incorporate ^15^N at the age of 10 weeks in several organs (e.g., liver, kidney, but not the brain) to successfully study protein expression levels in labeled vs. unlabeled individuals. To allow measurements of brain proteins, this method was improved in a recent study by McClatchy et al. [9] and successfully used in two further studies on rats [10], [11]. Though none of the studies using SILAM reported health problems or obvious behavioral changes, so far no studies have examined the potential effects of ^15^N incorporation into the proteome on the phenotype of the organism.

In the present study we tested various food compositions (blue-green algae vs. bacteria diet) and feeding protocols, and their effects on health and psychopathological relevant behaviors as well as on incorporation rate and data analysis in mice.

For our experiments, we used high-anxiety, “normal”-anxiety and low-anxiety mice of the HAB/NAB/LAB animal model for trait anxiety and co-morbid depression [12], [13]. The genetic predisposition of these animals was examined in multiple approaches ([14], [15]; Czibere et al., unpublished). In a 2D-gel study, glyoxalase-1 (GLO1) was identified as a biological marker being differentially expressed between the lines [12], [16]. However, taking the multifactorial and polygenic nature of emotional and psychopathological behavior into account, many more genes and their protein products are likely to contribute to the divergence of trait anxiety in this model and need to be identified.

In the present series of experiments, we describe the development of a highly efficient labeling method, based on a novel ^15^N-enriched bacteria diet using *Ralstonia eutropha* protein. This diet (developed in co-operation with Silantes GmbH, Germany) turned out to have superior methodological and analytical properties over the prior used ^15^N-enriched blue-green algae diet. Therefore, the new ^15^N bacteria diet allowed a highly sensitive MS analysis to compare the proteomes of HAB/NAB/LAB mice. As proof of concept, we show expression differences of potential biomarkers in cerebellar brain tissue, including GLO1, discovered in earlier studies, and histidine triad nucleotide binding protein 2 (Hint2), previously not found to be differentially expressed in the HAB/NAB/LAB model.

Additionally, we report for the first time a novel phenomenon of ^15^N enrichment having antidepressant-like effects in highly anxious animals. This is discussed as an avenue for the discovery of candidate proteins involved in depression-like behavior.

## Results

In preliminary studies we found that the offspring of CD1 dams fed exclusively with a blue-green algae diet had severe developmental problems that led to death by malnutrition at about 10 days after birth. By feeding the dams until weaning with a free choice of blue-green algae and standard diet, this problem was overcome. With animals preferring the blue-green algae diet four times over standard chow, this resulted in minor reduction of the incorporation rate only.

**Figure 1:**
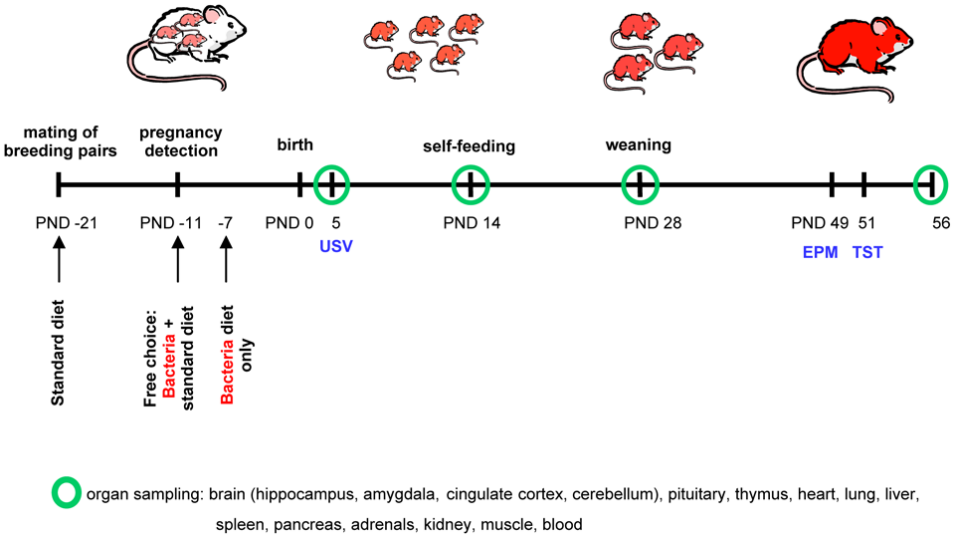
Mouse feeding protocol with the novel bacteria diet. As soon as pregnancy was detected 10 days after mating (post natal day PND -11), the animals received 4 days of *ad libitum* standard/bacteria diet before they were fed bacteria diet only. Organs were harvested at PND 5, 14, 28 and 56 to determine ^15^N incorporation rate and line-specific protein expression pattern.

The novel bacteria diet (Table 1) on the other hand had none of these shortcomings at any developmental stage. Feeding our animals with the bacteria diet exclusively after pregnancy detection and 4 days of free choice to accustom the animals to the new diet (Figure 1) was therefore considered the preferred method for achieving minimal food consumption and maximal ^15^N incorporation rates at early developmental stages.

**Table 1:** Bacteria and algae diet compositions.

Bacteria diet (Silantes GmbH, Germany)	Algae diet (Harlan Laboratories, USA)
20% Ralstonia eutropha (lyophilized 15N-labeled bacterial hydrolysate; isotope enrichment >98%))	33% Spirulina Powder (customer supplied)
35% Sucrose	27.5% Sucrose
13% Maltodextrin	10% Maltodextrin
10% Corn Starch	9.5% Vegetable Shortening (Primex)
0.3% L-Cystine	7.5% Anhydrous Milkfat
8% Soybean Oil	3% Soybean Oil
7.2% Cellulose	7.5% Cellulose
0.3% Calcium Phosphate, dibasic	0.6% Calcium Phosphate, dibasic
0.025% Ferric Citrate	0.6% Calcium Carbonate
1.4% Vitamin Mix, AIN-93-VX (94047)	0.25% Vitamin Mix, w/o choline, A, D, E (83171)
0.25% Choline Bitartrate	0.25% Choline Bitartrate
0.0024% THBQ, antioxidant	0.01% Vitamin E, DL-alpha tocopheryl acetate (500 IU/g)
4.5% Mineral Mix, AIN-93M-MX (94049)	0.00025% Vitamin D3, cholecalciferol (400,000 IU/g in sucrose)
	0.004% Zinc Carbonate
	0.001% Cupric Carbonate
	0.0002% Potassium Iodate

### Food Consumption and Weight Gain

**Figure 2:**
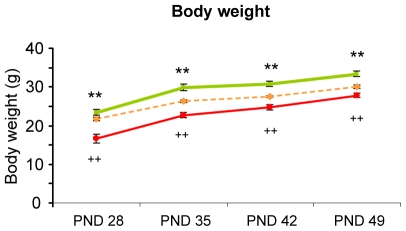
Body weight after weaning of animals fed with bacteria, blue-green algae and standard diet. Compared to standard fed HAB animals (orange dotted line), blue-green algae diet fed animals were significantly heavier (green line) and bacteria fed animals lighter (red line) (**p<0.01 blue-green algae vs. control ++p<0.01 bacteria vs. control).

No differences in overall food consumption of dams and the offspring before weaning were found for any mouse line, diet or feeding protocol (Table 2). However, whereas blue-green algae diet fed HAB animals showed a significantly increased body weight at weaning through to adulthood (p<0.01), bacteria diet fed animals had a reduced body weight compared to standard diet fed animals (p<0.01; Figure 2).

**Table 2:** Food consumption of algae and bacteria diets of HAB/NAB/LAB dams and pups before weaning.

Algae diet [g]					
Mouse Line Diet	HAB		
	14N	15N	Standard		
PND 1	8.0±0.5	7.4±0.9	9.2±0.7		
PND 7	11.5±0.6	11.0±0.7	13.7±0.5		
PND 14	13.9±0.4	12.1±1.0	15.4±0.9		
PND 24	15.8±2.0	15.4±1.8	18.3±1.0		

Bacteria diet [g]					
Mouse Line Diet	HAB	NAB	LAB
	14N	15N	14N	15N	14N
PND 1	4.5±0.4	5.8±0.6	8.0±2.0	6.0±0.7	6.2±0.7
PND 7	8.9±1.0	8.1±1.4	11.1±1.6	11.6±1.4	9.3±1.1
PND 14	9.9±0.8	8.4±0.6	14.6±1.8	11.7±1.1	10.5±1.3
PND 24	13.1±1.9	11.9±1.2	17.4±4.9	11.7±2.0	14.0±1.9

Consumption of blue-green algae diet of HAB dams and their pups and consumption of bacteria diet of HAB/NAB/LAB dams and their pups before weaning (post natal days (PND) 1–24); ^14^N diet 14N; ^15^N-enriched diet.

### ^15^N Incorporation Rate

**Figure 3:**
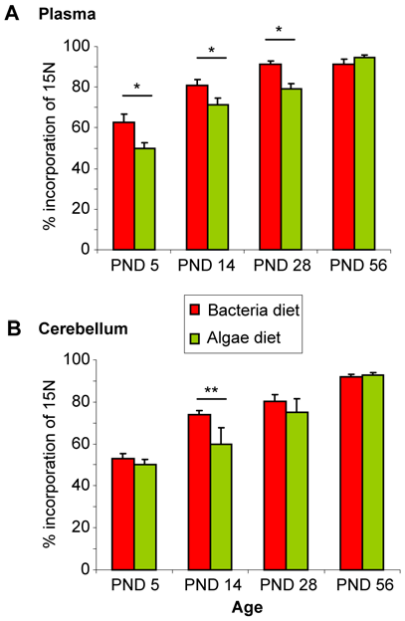
Incorporation rate of ^15^N in (A) plasma and (B) brain proteins. In 5, 14 and 28 day old animals (PND 5, 14, 28) bacteria diet feeding provides a significantly higher incorporation of ^15^N in plasma proteins compared to blue-green algae diet; only at the age of 56 days, blue-green algae fed diet animals show the same ^15^N incorporation. In the cerebellum, a faster incorporation in adolescence at PND 14 reached significance (**p<0.01; *p<0.05).

The incorporation rate of ^15^N in plasma proteins was significantly higher in bacteria compared to blue-green algae diet fed animals on post natal days (PND) 5, 14 and 28 (p<0.05; Figure 3). While the cerebellum had a relatively low incorporation rate in animals fed with any ^15^N diet on PND5, bacteria fed animals showed an earlier increase of ^15^N incorporation in adolescence compared to blue-green algae fed animals (p<0.01). At PND56, all animals approached a maximal incorporation rate of over 90% in all tissues independent of the diet used.

### Anxiety-Related and Depression-Like Behaviors

**Figure 4:**
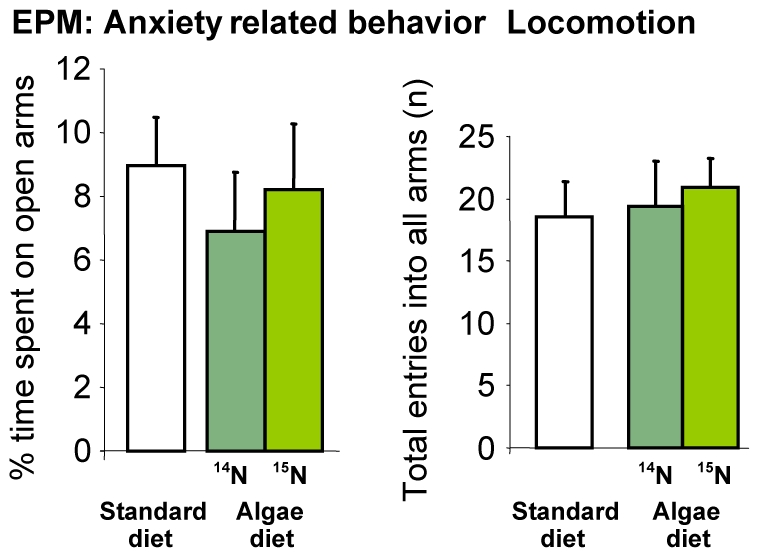
Anxiety-related behavior and locomotion of HAB animals on the elevated plus maze (EPM). No behavioral changes due to the blue-green algae diet fed to the animals were found.

**Figure 5:**
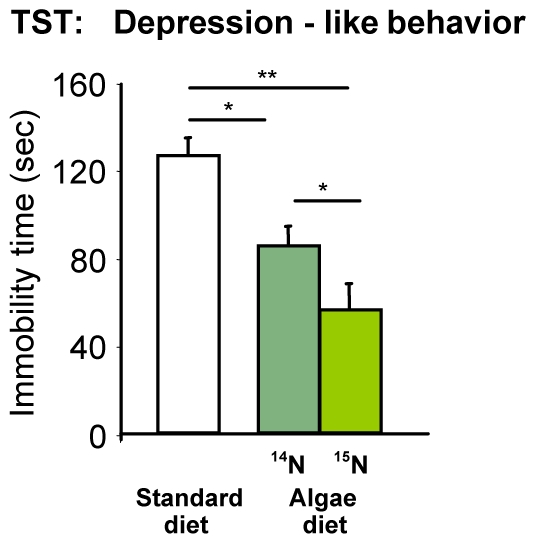
Depression-like behavior of HAB animals in the tail suspension test (TST). Animals fed with blue-green algae diet hat a strongly reduced immobility, indicating depression-like behavior, compared to standard fed animals. This effect was even more pronounced in animals fed with the ^15^N isotope (*p<0.05; **p<0.01).

The blue-green algae diet, when fed to HAB animals, had no impact on anxiety-related parameters or locomotion (Figure 4). However, HAB animals fed with a blue-green algae diet showed a strongly reduced time of immobility in the tail suspension test compared to standard diet fed animals, indicative of diminished depression-like behavior (p<0.05), which was even more pronounced in ^15^N diet fed animals (p<0.01; Figure 5).

**Figure 6:**
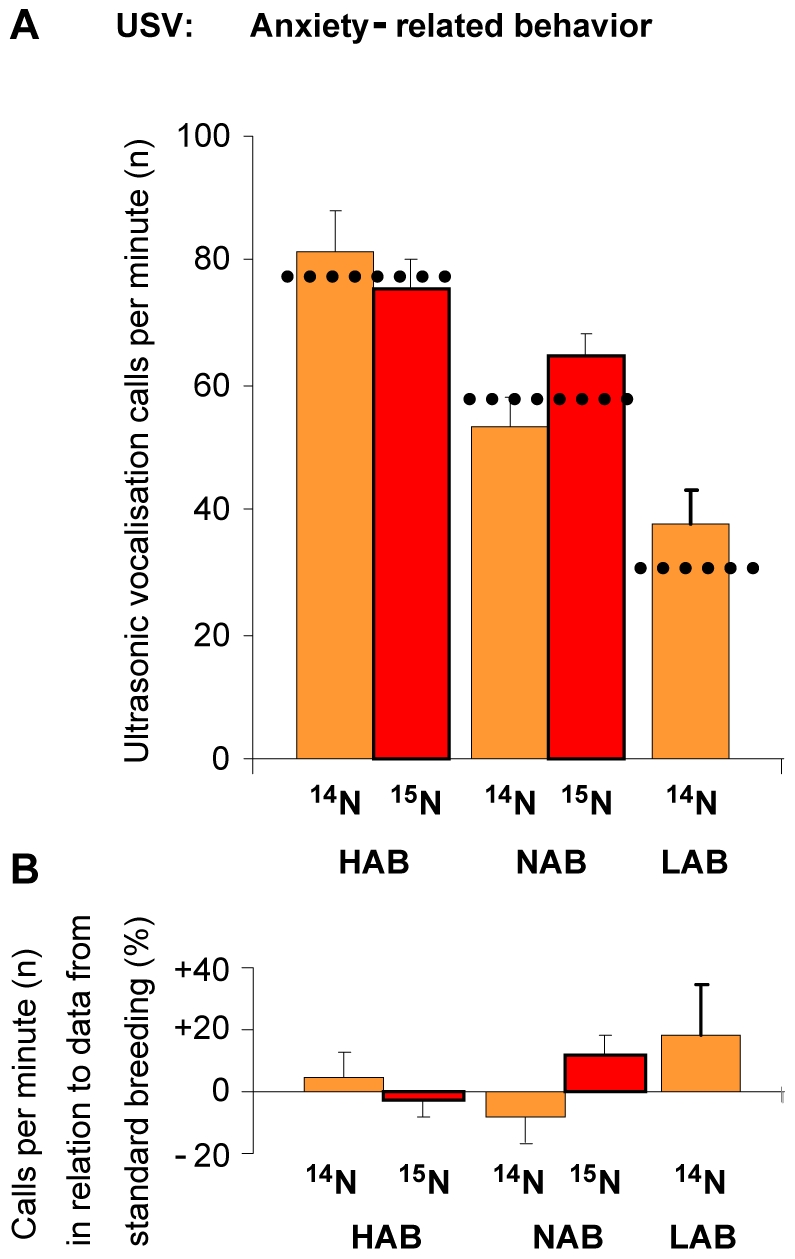
(A) Anxiety-related behavior at post natal day 5 in the ultrasonic vocalization test (USV). High (HAB), normal (NAB) and low (LAB) anxious animals were fed with ^14^N or ^15^N enriched bacteria diets. **A**) Independent of the diet, HAB, NAB and LAB mice showed the significant (not indicated) phenotypic divergence typical of animals of the standard breeding (dotted lines and indicated in **B**) as percentage difference of the standard breeding).

**Figure 7:**
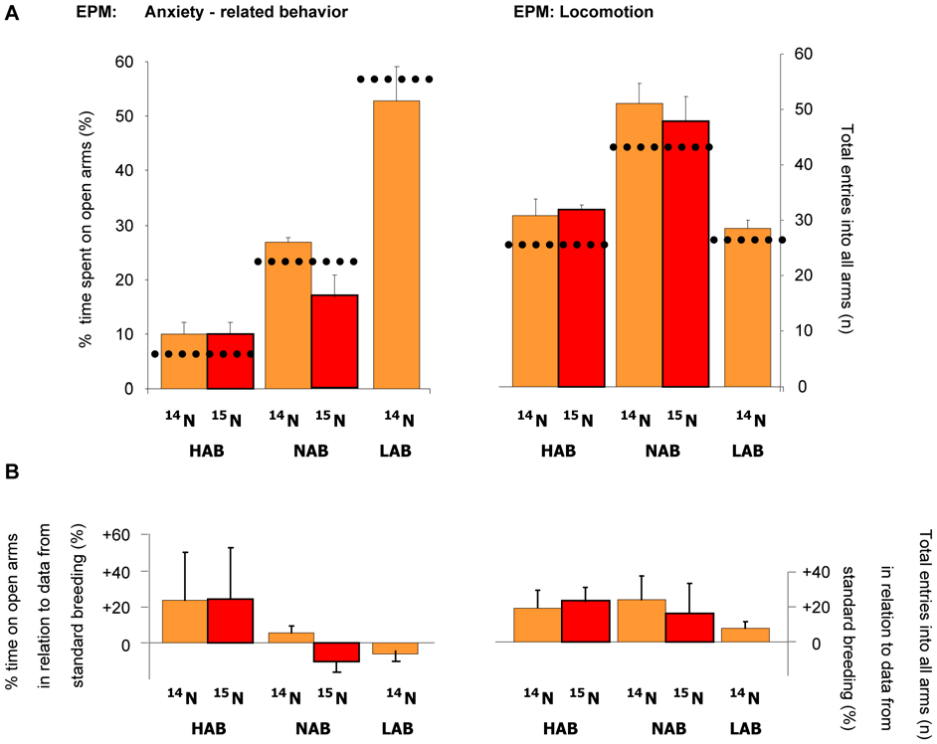
Anxiety-related behavior and locomotion on the elevated plus maze (EPM). High (HAB), normal (NAB) and low (LAB) anxious animals were fed with ^14^N or ^15^N enriched bacteria diets. **A**) HAB, NAB and LAB mice showed the significant (not indicated) phenotypic divergence typical of animals of the standard breeding (dotted lines and indicated in **B**) as percentage difference of the standard breeding).

The bacteria diet, similar to the blue-green algae diet, had no influence on anxiety-related behaviors or locomotion of HAB, NAB and LAB mice as they showed the same significant phenotypic divergence typical of animals of the standard breeding in both the ultrasonic vocalization and the elevated plusmaze tests (Figures 6 and 7; data from HAB/NAB/LAB standard breeding generations 26–29 in Figure 6A as dotted line and depicted in Figure 6B as percentage difference compared to experimental animals: Kessler, Bunck and Frank, unpublished data; [12], [13], [16], [17]).

**Figure 8:**
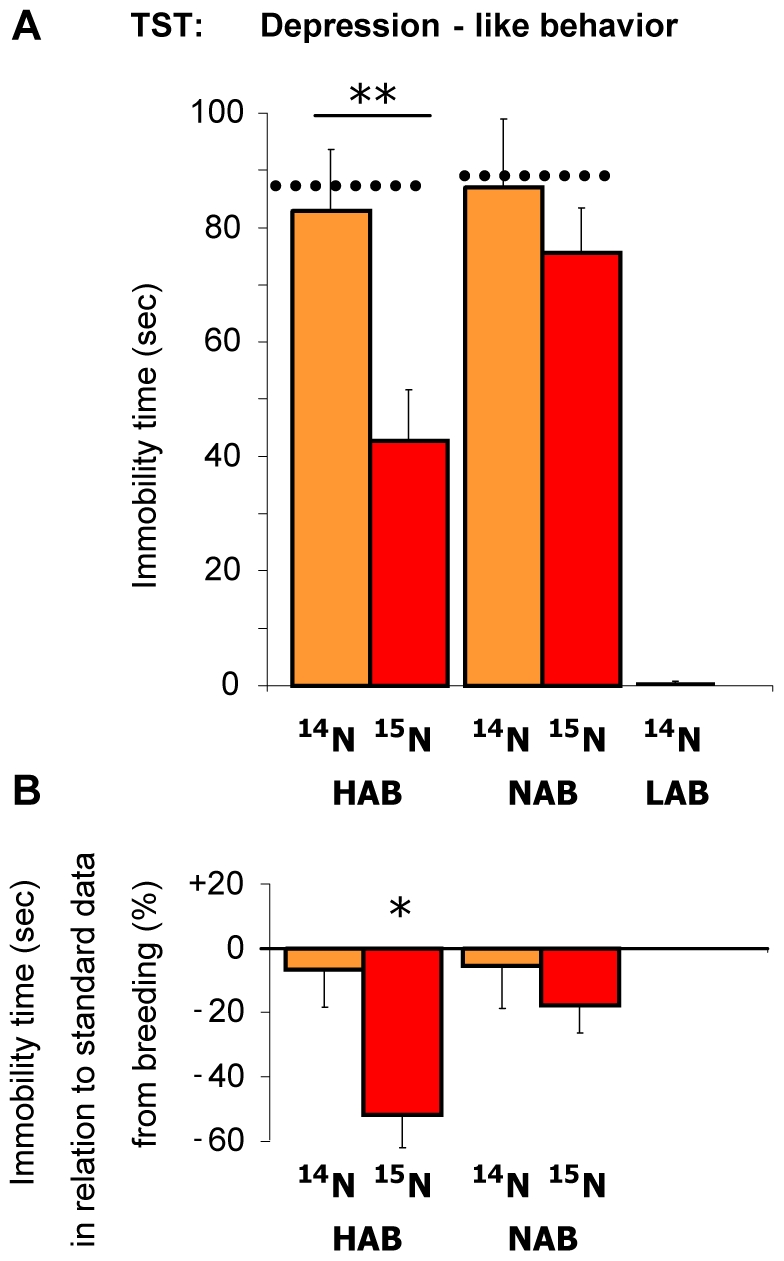
Depression-like behavior in the tail suspension test (TST). High (HAB), normal (NAB) and low (LAB) anxious animals were fed with ^14^N and/or ^15^N enriched bacteria diets. **A**) No behavioral changes due to diet *per se* were found for animals fed with bacteria diet compared to the animals of the respective lines of the HAB/NAB/LAB standard breeding of the same generation (dotted lines and indicated in **B**) as percentage difference of the standard breeding). However, ^15^N fed HAB animals showed a strongly reduced immobility, indicating depression-like behavior, compared to ^14^N fed HABs (**p<0.01). B) This differed significantly from the standard HAB/NAB/LAB breeding (*p<0.05). Due to division by zero, no value is given for ^14^N fed LAB animals in relation to the standard breeding: values are ^14^N LAB 0.53±0.3 sec vs. standard LAB 4.06±1.5 sec.

In contrast to the blue-green algae diet, the bacteria diet *per se* had no influence on depression-like behavior in the TST (Figure 8). However, confirming the data gained from the blue-green algae diet, feeding HAB animals with the stable ^15^N isotope using the bacteria diet resulted in a strong decrease in the time of immobility compared to animals fed with ^14^N bacteria diet (p<0.01), reflecting the animals' depression-like behavior (Figure 8). Whereas ^15^N-fed HAB animals still showed considerably more depression-like behavior than LAB animals (p<0.01; Figure 8), these data demonstrate a clear diet-independent antidepressant-like effect of the ^15^N isotope in high-anxiety mice.

### Protein Expression Analyses by MS

**Figure 9:**
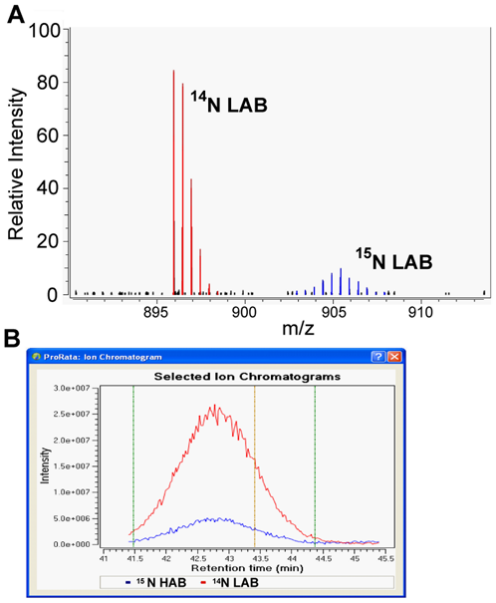
Analysis of expression differences by MS analysis. **A**) ESI mass spectrum of the ^14^N LAB (red) and ^15^N HAB (blue) isotope forms of the GLO1 peptide GFGHIGIAVPDVYSACK in cerebellar tissue. The isotopologue patterns of the ^14^N and ^15^N peptide signals (m/z) were used for relative quantification with ProRata. **B**) Extracted ion chromatograms for the GLO1 peptide, extracted from A, showing an upregulation of GLO1 in LAB mice. No smoothing was applied.

**Figure 10:**
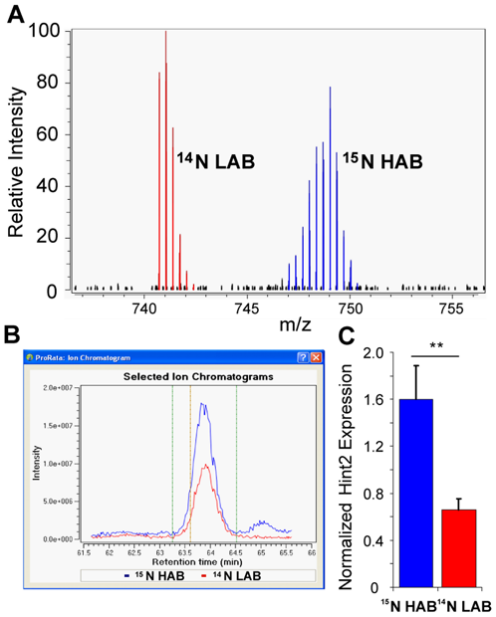
Analysis of expression differences by MS analysis and RT-PCR. **A**) ESI mass spectrum of the ^14^N LAB (red) and ^15^N HAB (blue) isotope forms of the Hint2 peptide ISQAEEDDQQLLGHLLLVAK in cerebellar tissue. The isotopologue patterns of the ^14^N and ^15^N peptide signals (m/z) were used for relative quantification with ProRata. **B**) Extracted ion chromatograms for the Hint2 peptide, extracted from A, showing an upregulation of Hint2 in HAB mice. **C**) Analysis of Hint2 transcripts in ^15^N-labeled HAB and ^14^N LAB cerebellar cDNA by RT-PCR, normalized by HRSP12 expression. Hint2 expression in HABs was 2.5-fold higher compared to LABs (*p<0.05). No smoothing was applied.

Examination of GLO1 expression by targeted MS analysis verified the well-established difference in protein expression between HAB and LAB animals [12], [13]. Quantification of protein abundance in cerebellar tissue revealed, after normalization, a significant 3.9-fold increase of GLO1 in ^14^N labeled LABs compared to ^15^N labeled HABs (Figure 9A, B). In addition to this “standard marker”, Hint2 could be identified as differentially expressed in HAB vs. LAB animals (Figure 10A, B). The analysis of cerebellar tissue of ^15^N-labeled HAB vs. ^14^N-labeled LAB animals using ESI MS revealed a significant 2.4-fold increase in Hint2 protein expression in HAB mice, as shown by Hint2 tryptic peptide quantification. This difference in Hint2 expression was confirmed by RT-PCR in unlabeled animals of both lines, with Hint2 being 2.5-fold more abundant in HAB compared to LAB cerebellar cDNA (Figure 10C).

## Discussion

We demonstrate that ^15^N labeling of mice via a bacteria diet is a feasible and cost-effective method to study the proteomic differences related to psychopathologies in the HAB/NAB/LAB animal model using MS. Whereas the novel bacteria diet *per se* had no effect on anxiety- and depression-like endophenotypes of the animals, the stable isotope ^15^N repeatedly decreased depression-like behavior in high anxious animals, irrespective of the diet used (Figures 5 and 8). We are not aware of any other study showing that a stable isotope can affect behavioral characteristics of labeled animals. A possible explanation for this phenomenon could be altered enzymatic activities of ^15^N-labeled proteins due to increased chemical bond strength, which might affect pathways involved in the pathobiology of anxiety/depression. Indeed, Ditzen et al. [14] have recently described altered enzyme kinetics likely contributing to the phenotype of HAB animals. Altogether, the phenomenon of alterations in depression-like behavior due to ^15^N labeling, with the anxiety-related behavior remaining unchanged, might allow us to carry out further analyses to detect critical, so far unstudied, mechanisms exclusively involved in the depression-like behavior of HAB mice. Additionally, these results clearly demonstrate the necessity to confirm the behavioral phenotype of a mouse model fed with a ^15^N diet.

Metabolic labeling of non-mammalian organisms with ^15^N for comparing two proteomes by MS analysis is a valuable technique and routinely used in quantitative proteomic approaches [4]. Labeling rats with a ^15^N-enriched blue-green algae diet has demonstrated that the method is applicable to rodents [8], [9]. However, studying psychopathologically relevant proteomic parameters requires the examination of any diet effect *per se* onto relevant behaviors. For ^15^N-labeled rats fed with a blue-green algae-based diet, no obvious behavioral changes were reported. However, no thorough phenotypic analysis of the animals was reported [8]. Furthermore, the usage of a restricted feeding paradigm does *a priori* not allow the exclusion of an impact on the animals' stress-related behaviors. This was solved by using blue-green algae powder incorporated in normal pellets fed *ad libitum* [9]. To be able to correlate psychopathological behavioral parameters with protein levels as early as PND5 via ultrasound vocalization, it was necessary to use a feeding protocol, in which the pregnant dams were already fed with the respective diet. As reported by McClatchy et al. [9], a low incorporation rate of ^15^N is expected in slow turnover tissues, like the brain, compared to rapidly turnover tissues, like the liver. Indeed, the authors succeeded in presenting a feeding protocol for rats that started with the pregnancy of the dam, which provided an incorporation rate of up to 94% for all body tissues, but is quite cost-intensive. However, whereas rats did not seem to have any problems coping with blue-green algae enriched diet over a whole generation, preliminary studies in our laboratory indicated that the offspring of mice exclusively fed with blue-green algae diet had severe developmental problems.

We were able to overcome the problem of malnutrition in early development by free choice blue-green algae diet/standard chow feeding. This resulted in a sufficient incorporation rate of ^15^N in all tissues in adulthood. However, the incorporation rate in adolescence was considerably low and, more importantly, we observed a strong impact of the blue-green algae diet *per se* on depression-like behavior of the adult animals. This effect was even more pronounced in animals fed with ^15^N-enriched blue-green algae diet.

We therefore developed (in co-operation with Silantes GmbH, Germany) a novel diet based on *Ralstonia eutropha* bacteria, which we found to be not only better tolerable for the animals but also more cost-effective. Indeed, using this diet, we did not encounter any health problems of the dams or the offspring. Only the weight of the animals was slightly reduced when fed with the bacteria diet (Figure 2). This had, however, no detectable phenotypic consequences. Whether the reduced weight was due to the slightly different diet composition compared to standard lab chow or the addition of bacteria remains to be shown. The incorporation rate of ^15^N was found to be over 60% as early as PND5, when divergent anxiety-related behavior is clearly detectable for the first time, and significantly higher than in free choice blue-green algae diet fed animals (Figure 3). This will be advantageous for future studies on the ontogenetic development of complex traits such as anxiety-related behavior. Even more important, no influence of the bacteria food *per se* on any of the examined behaviors of HAB/NAB/LAB mice was found. Indeed, although the ^15^N isotope had an impact on the behavior of the animals, as discussed earlier, their phenotypes were still highly divergent. This demonstrates the advantage of using highly selected animals with extremely divergent phenotypes for labeling studies to allow strong correlations between endophenotypes and proteomic differences, taking a possible effect of ^15^N into account.

In highlighting two differentially regulated proteins, we validated the method and demonstrated that it has the potential to identify novel phenotype-related protein expression differences. The standard biomarker of the HAB/NAB/LAB animal model, GLO1 [12], [16], was successfully detected and validates the ^15^N approach used in this study. Additionally, Hint2 was found to be differentially expressed between HAB and LAB mice. This enzyme catalyses hydrolysis and transfer of nucleotide-containing substrates in metabolic pathways of DNA, RNA and carbohydrates. Located in the mitochondrial membrane, it is thought to maintain mitochondrial potential and calcium release, thereby influencing mitochondria-dependent apoptosis. Moreover, Hint2 was shown to impact on endocrine factors by acting on steroidogenesis in H295R cells [18]. While more conventional 2D-gel analyses used in prior studies failed to detect a difference in Hint2 (18 kDa), the present approach allowed the detection of candidate proteins with both low and high molecular weights. Indeed, RT-PCR and recent microarray data confirm the differential expression of Hint2 in HAB vs. LAB mice (Figure 10C and Czibere et al, unpublished).

Therefore, labeling animals with ^15^N via the novel bacteria diet, verifying their phenotype and examining highly sensitive divergences in their proteomes due to both ^15^N incorporation and genetic predisposition will open up new dimensions of detection of proteins and pathways underlying psychopathological traits.

## Materials and Methods

All diets were prepared as pellets (either by Harlan Laboratories or Silantes GmbH, Germany) with comparable size and consistency to standard lab chow.

### Diet Compositions

The composition of the bacteria and blue-green algae diet are shown in table 1. Standard laboratory diet (Harlan Laboratories, Germany) was used as control diet.

### Animals

HAB, NAB and LAB mice, bred and housed in the animal facilities of the Max Planck Institute of Psychiatry (Munich, Germany) were used in this study. The mouse lines are derived from CD1 and were inbred for extremes in trait anxiety according to their anxiety-related behavior on the elevated plus maze (EPM), with HAB mice spending <10%, NAB mice spending approx. 30%, and LAB mice spending >50% on the open arms of the EPM [12]. Dams were housed in type 3 macrolone cages, mature animals were housed in groups of 4 animals in type 2 macrolone cages (12 h light/dark cycle (lights on at 6 am), room temperature 23±2°C, humidity 60%; tap water and food *ad libitum*). Behavioral experiments were performed between 8 am and 1 pm.

The presented work complies with current regulations covering animal experimentation in Germany and the EU (European Communities Council Directive 86/609/EEC). All experiments were announced to the appropriate local authority and were approved by the ‘Animal Welfare Officer’ of the Max Planck Institute of Psychiatry.

### Behavioral Tests

#### Ultrasonic vocalization test

On PND 5, pups were individually separated from their mothers and immediately placed on a Petri dish (∅ 15 cm, cleaned with 70% ethanol; temperature was kept constant by water-bath (23°C) underneath the dish). USV calls were detected and counted for 5 min with a bat detector (Mini 3 bat-detector, Ultra Sound Advice, U.K.) at 70 kHz.

#### Elevated plus maze test

The plus-shaped maze is built of dark gray PVC and consists of two respectively opposing open (30×5 cm, 300lux, white light) and closed arms (30×5×15 cm, 10lux, red light) connected by a central platform (5×5 cm, 90lux), located 40 cm above the floor, surrounded by a black curtain and was cleaned before each trial with water containing a detergent [19]. The animal's behavior was recorded for 5 min via a video camera fixed above the maze. Percentage of time spent on the open arms relative to the time spent on all arms and the total number of entries into all arms were monitored by a trained observer blind to treatment or tracking software.

#### Tail suspension test

For this test, the mouse was suspended by the end of its tail to a bar 35 cm above the floor [20]. The animals' behavior was videotaped for 6 min and the duration of total immobility scored by a trained observer blind to the treatment.

### Experimental Procedure (Figure 1)

To gain a high incorporation rate of ^15^N at early developmental stages, mice were fed with ^15^N-enriched diet from embryonic status on. As controls, mice received according to the same protocol ^14^N non-labeled diet or standard chow. Females (4–8 per diet) were mated one-to-one with an adequate male (i.e. sibling) to increase the pregnancy probability and to gain the optimal number of required offspring. After 10 days, the females' pregnancy state was estimated visually and/or by palpation of the embryos along the backbone, and the males were removed. Pregnant dams were fed for 4 days *ad libitum* with both standard and the respective special diet to habituate to the new diet. Then the animal was supplied with pure special diet.

On PND5, all offspring was tested for USV and litters got culled to an average number of the usual litter size. On day 28, animals were weaned and group housed (2–4 animals per cage). Each cage was supplied with the respective special diet in accordance to the mother's diet. Groups were composed of animals derived evenly from all litters per diet to minimize cage effects. At 7 weeks of age, all animals were tested in the elevated plusmaze test and 48 h later in the tail suspension test. Tissue from all animals was collected one week after the last behavioral test after perfusion with 0.9% saline. Blood was sampled before the perfusion procedure and centrifuged at 13000 g for 10 min. Plasma and all collected tissue were snap frozen and stored at −80°C.

### ^15^N incorporation Rate Determination

To monitor the ^15^N incorporation throughout the breeding scheme, ^15^N incorporation rates were calculated for PND5, 14, 28 and 56 in brain and plasma samples. For every time point, up to four pairs of ^15^N and ^14^N mouse cerebella were mixed at a ratio of 1∶1 (w/w). Tissue was homogenized in 250 mM sucrose (Sigma Aldrich, St. Louis, MO, USA) buffer containing 50 mM Tris-HCl, 5 mM MgCl_2_, 1 mM DTT (BioRad, Hercules, CA, USA), spermine (25 ug/ml, Sigma), spermidine (25 ug/ml, Sigma) and Cocktail inhibitor tablets (Roche Diagnostics, Indianapolis, IN, USA) and centrifuged at 25000 g, for 1 h at 4°C. The supernatant was concentrated at 13000 g, for 30 min at 20°C using 3kDa cut off spin filters (Millipore, Bellerica, MA, USA), the extracted protein mixture was resolved by 12.5% SDS-PAGE and the gel stained with Coomassie blue (BioRad). Several abundant gel bands were chosen, cut into small pieces, washed twice with 25 mM NH_4_HCO_3_/50% acetonitrile (VWR, Darmstadt, Germany) and digested with trypsin (5 ng/ul, Promega, Madison, WI, USA) overnight at 37°C. Peptides were extracted with 50% acetonitrile/2% HCOOH (Merck, Darmstadt, Germany), lyophilized, redissolved in 10 µl 0.5% trifluoroacetic acid (Sigma), cleaned up by OMIX tips (Varian, Palo Alto, CA, USA) and then analyzed with a MALDI-TOF-TOF Ultraflex II mass spectrometer (Bruker Daltonik, Bremen, Germany). MS/MS data were searched against a Swissprot 51.6 mouse database using MASCOT 2.2 (Matrix Science, London, UK). The ^15^N incorporation rates of the labeled fractions were determined by the in-house software *QuantiSpec* [21]. For determining ^15^N incorporation in plasma, ^15^N and ^14^N mouse plasma proteins were mixed at a 1∶1 ratio (v/v) and processed as described for the brain samples.

### Relative Quantification

To compare the protein profiles of HAB and LAB animals, ^15^N labeled cerebella from HAB mice were compared with ^14^N cerebella from LAB mice. Three ^15^N HAB/^14^N LAB cerebella pairs were compared. Per pair, a ^15^N cerebellum was combined with a ^14^N cerebellum at a 1∶1 ratio (w/w). Cytosolic proteins were then extracted according to the protocol of Cox and Emily (Cox and Emily, 2006). Protein content was estimated with Bradford assay (BioRad) and 100 µg per ^14^N/^15^N pair were loaded onto a 12.5% SDS PAGE. For protein digestion gel bands were washed twice with 25 mM NH_4_HCO_3_/50% acetonitrile (VWR), followed by reduction with 10 mM dithiothreitol (BioRad) for 30 min at 56°C, carboxyamidomethylation with 50 mM iodoacetamide (BioRad) for 30 min at room temperature and subjected to digestion with trypsin (5 ng/ul, Promega, Madison, WI, USA) overnight at 37°C. Peptide extraction was performed as described above. For every fraction, peptides were lyophilized and dissolved to 10 µl 1% HCOOH (Merck). Five µl were then loaded onto an in-house packed fused silica RP-C18 (3 µm, Maisch, Monheim, Germany) column (0.075 mm×20 cm), washed with 0.1% HCOOH (Merck) for 20 min and eluted with a gradient of 95% acetonitrile/0.1% HCOOH (Merck) over 120 min at a flow rate of 200 nl/min using a nanoLC-2D system (Eksigent, Dublin, CA, USA). Column effluents were directly infused into an LTQ-Orbitrap mass spectrometer (Thermo Fisher Scientific, Bremen, Germany). The five most abundant precursor ions in the full scan were subjected to MS/MS fragmentation (‘Top Five’). Mass spectrometry RAW files were searched against a ^14^N and a ^15^N decoy ipi.MOUSE.v3.46 database utilizing BioWorks 3.3.1 and SEQUEST software (Thermo Fischer Scientific, San Jose, CA, USA). Precursor and fragment ion tolerance were set to 5ppm and 1Da, respectively. Trypsin was chosen as enzyme and up to two missed cleavage sites were allowed. Cysteine carboxyamidomethylation was used as static modification. Methionine oxidation was used as variable modification. Quantification was performed using the ProRata 1.0 software (Pan et al., 2007).

### Real Time PCR

Total RNA was isolated from mouse cerebellar brain tissue, using Tri reagent (Ambion, Huntingdon UK) and reverse-transcribed using oligo-dT-primers. cDNA was precipitated in 1 M sodium acetate and 60% ethanol in liquid nitrogen. Twenty ng of purified cDNA were analyzed in a reaction volume of 10 µl, using the QuantiFast SYBR Green PCR Mix (Quiagen, Hilden Germany) and a capillary Light Cycler 2.0 (Roche Diagnostics, Mannheim Germany). cDNA of each brain was quantified in duplicates. For quantification of Hint2-transcripts, primers Hint2F (aggacacttactccttgtggc) and Hint2R (caagtacgtgaatgtgcagg) were used. Signal intensities were normalized by the amount of heat-responsive protein 12 (Hrsp12) housekeeper, found in individual brain samples, using primers HRSP12F (atgaatgactttggcactgtc) and HRSP12R (atggctacttatagtcatgcc).

### Statistical Analysis

The data are presented as means±SEM and were statistically analyzed using SPSS 12.0. Data dependent on two or more variables were compared using univariate analysis of a general linear model, subsequently split and further analyzed by T-test. Bonferroni correction was applied to adjust for multiple comparisons. Significance was accepted with p<0.05.
